# Kinematic, Neuromuscular and Bicep Femoris In Vivo Mechanics during the Nordic Hamstring Exercise and Variations of the Nordic Hamstring Exercise

**DOI:** 10.3390/muscles3030027

**Published:** 2024-09-18

**Authors:** Nicholas Ripley, Jack Fahey, Paul Comfort, John McMahon

**Affiliations:** 1 School of Health and Society, University of Salford, Salford M5 4WT, UK; j.t.fahey@salford.ac.uk (J.F.); p.comfort@salford.ac.uk (P.C.); j.j.mcmahon@salford.ac.uk (J.M.); 2 School of Medical and Health Sciences, Edith Cowan University, Joondalup 6027, Australia; 3 Hawkin Dynamics, Westbrook, ME 04092, USA

**Keywords:** fascicle length, dynamic ultrasound, eccentric, isometric, hamstring, injury risk

## Abstract

The Nordic hamstring exercise (NHE) is effective at decreasing hamstring strain injury risk. Limited information is available on the in vivo mechanics of the bicep femoris long head (BF_LH_) during the NHE. Therefore, the purpose of this study was to observe kinematic, neuromuscular and in-vivo mechanics of the BF_LH_ during the NHE. Thirteen participants (24.7 ± 3.7 years, 79.56 ± 7.89 kg, 177.40 ± 12.54 cm) performed three repetitions of the NHE at three horizontal planes (0°, 20° and −20°). Dynamic ultrasound of the dominant limb BF_LH_, surface electromyography (sEMG) of the contralateral hamstrings and sagittal plane motion data were simultaneously collected. Repeated measures analysis of variance with Bonferroni post hoc corrections were used on the in vivo mechanics and the kinematic and sEMG changes in performance of the NHE. Likely differences in ultrasound waveforms for the BF_LH_ were determined. Significant and meaningful differences in kinematics and in vivo mechanics between NHE variations were observed. Non-significant differences were observed in sEMG measures between variations. Changes to the NHE performance angle manipulates the lever arm, increasing or decreasing the amount of force required by the hamstrings at any given muscle length, potentially changing the adaptive response when training at different planes and providing logical progressions ore regressions of the NHE. All NHE variations result in a similar magnitude of fascicle lengthening, which may indicate similar positive adaptations from the utilization of any variation.

## 1. Introduction

The structure of the hamstring muscles, including fascicle length (FL), adapts in varying ways in response to different training stimuli, which is considered a crucial element in the reduction in hamstring strain injury (HSI) risk [1]. Supramaximal eccentric exercises, such as the Nordic hamstring exercise (NHE), have been shown to increase bicep femoris long head (BF_LH_) FL [2]. This contrasts with quasi-isometric (e.g., razor curls) and short muscle length conventional hamstring training (e.g., lying leg curls), where researchers have reported no change and a decrease in BF_LH_ FL, respectively [1,3,4]. However, to date, research is limited in quantifying the muscle fascicle dynamics during hamstring resistance exercises [5,6]. Such information may aid researchers and practitioners in explaining why preferential adaptations (i.e., increased BF_LH_ FL), may occur when utilizing the NHE.

There is a consensus on the complexity of collecting dynamic ultrasound on the BF_LH_ [5,6]. More recently, researchers have been successful at imaging the BF_LH_ fascicles during various exercises, including the NHE [5,6]. Raiteri et al. [5] observed lengthening of the BF_LH_ FL changes during the NHE. From 85% of peak force to peak force attained during the NHE there was significant lengthening of the BF_LH_ FL (from 4.2 ± 2.7 mm to 8.1 ± 5.5 mm, dependent on the tracking method). The sole study to identify in vivo muscle mechanics across the entire NHE, Van Hooren et al. [6] observed BF_LH_ FL changes during the NHE, while also observing single-leg Romanian deadlift and Roman chair hold. For the NHE, BF_LH_ FL was observed to initially shorten, but a lengthening was found to occur only after reaching the break point, with a 28.6 ± 10.9 mm excursion, potentially illustrating that the NHE is not actually an eccentric exercise. Fascicle changes within the single-leg Romanian deadlift followed and lengthening followed by shortening during hip flexion and hip extension, respectively, while, to no surprise, a Roman chair hold resulted in minimal change in BF_LH_ FL except at the end of the movement during the barbell lowering phase [6]. However, to date, no study has attempted to identify any changes in BF_LH_ FL during variations of the NHE, with consideration of progressing and regressing the exercise.

Implementation of the NHE within sports typically prescribes it as a supra-maximal exercise on a horizontal plane (0°). With continued application, there will be progressive overload by reaching a greater knee extension angle before reaching a break point, resulting in a greater moment arm; however, this should be followed with the progressive application of load, if full knee extension can be achieved under control, to continually see adaptations in risk factors of HSI (i.e., eccentric strength and BF_LH_ FL) [1,3,4]. Commonly, this progression includes the addition of external weight (2.5–5 kg); however, as the NHE is still not commonplace within both male and female elite sports [7,8], the addition of load could be a stumbling block in its application. Performing the NHE on an incline or decline would manipulate the lever through which the center of mass from the knee up is acting, thereby increasing or decreasing the amount of force required by the hamstrings to control the descent, until a break point is achieved when the hamstrings can no longer resist the lengthening, which can occur at any knee joint angle. To date, only a single study has been published identifying no differences in peak torque at the knee between neutral performance at 0°, 20° and 40° of inclinations, albeit with reduced muscle activity at greater knee extension angles [9]. However, a decline NHE, which should increase the peak torques and result in more acute knee extension angles, has not been observed. Therefore, the aims of this study were to investigate the angle at which the NHE is performed and its influence on the kinematic, neuromuscular and in vivo dynamics of the BF_LH_ throughout the movement. It was hypothesized that an incline NHE would reduce the intensity (i.e., decreased surface electromyography (sEMG) and increased break point angle), while a decline NHE would increase the intensity (i.e., increased sEMG and decreased break point angle). It was also hypothesized that the differences in the kinematics (knee extension angle) would result in comparable differences in in vivo fascicle lengthening.

## 2. Materials and Methods

Thirteen physically active individuals (10 males and 3 females, age 24.7 ± 3.7 years, body mass 79.56 ± 7.89 kg, height 177.40 ± 12.54 cm) with no history of lower-limb injury participated in this observational study, with participants chosen based on convenience sampling. All participants reported that they were physically active, with a previous history of performing the NHE within training and familiarity with the exercise, representing tiers 1 and 2 from the classification from McKay et al. [10]. Written informed consent was obtained from all participants prior to testing, citing no previous hamstring strain injury in the preceding six months. The study was approved by the ethics committee (HSR1819-048) and conformed to the principles of the Declaration of Helsinki (2013). A priori sample size estimation was performed using an average identified effect size (*d* = 0.8) between variations of the NHE in torque and kinematics from Sarabon et al. [9]; with a statistical power of 0.80 and an alpha error probability of 0.05 a projected sample of 10 was needed.

**Figure 1:**
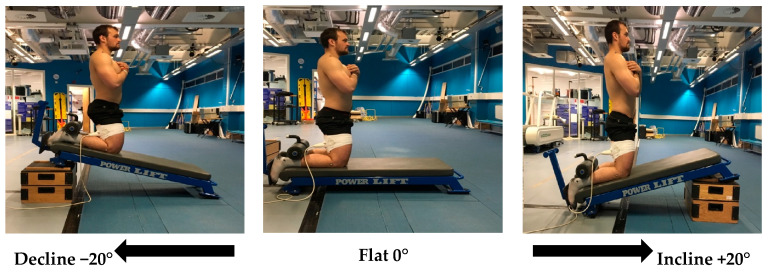
Nordic hamstring exercise variations: flat (0°), decline (−20°) and incline (20°).

Prior to performing the NHE, resting BF_LH_ architectural data were collected, followed by performance of a standardized warmup consisting of two sets of ten repetitions of bodyweight squats, lunges and leg swings. To perform the NHE, participants knelt on a padded Nordic bench (Power Lift, Jefferson, IA, USA), with the ankles secured immediately superior to the lateral malleolus by ankle pads that were secured to the bench. From the initial kneeling position, with arms held across the chest and hips extended, participants lowered their body as slowly as possible to a prone position. Participants only performed the lowering portion of the exercise, aiming to lower with as much control as possible, until the lowering phase could no longer be controlled and they reached a break point [11,12], at which point they were instructed to use their arms to control the decent in a push-up motion. The Nordic bench was positioned across 3 horizontal planes for NHE performance angles of flat at 0°, incline at 20° and decline at −20° (Figure 1) [9]. Participants were instructed to perform three repetitions of each variation in a random order, with a one-minute rest provided between each repetition and 2–3 min between each variation. Hip flexion was not permitted during the NHE and counted as a failed repetition; if hip extension was observed by the assessor, the repetition would be discounted and repeated after 2–3 min rest.

Resting BF_LH_ FL was collected with participants lying in a prone position with full knee and hip extension. Images were captured at the halfway point between the ischial tuberosity and the lateral epicondyle, along the line of the BF_LH_ in accordance with previous research [13,14]. A 10 cm linear array ultrasound probe with a layer of conductive gel was applied, with a depth resolution of 67 mm and a density of 48% (MyLab, Esaote, Genoa, Italy). Care was taken to ensure minimal pressure was applied to the skin, and the assessor (NJR) manipulated the orientation of the probe slightly if the superficial and intermediate aponeuroses were not parallel.

**Figure 2:**
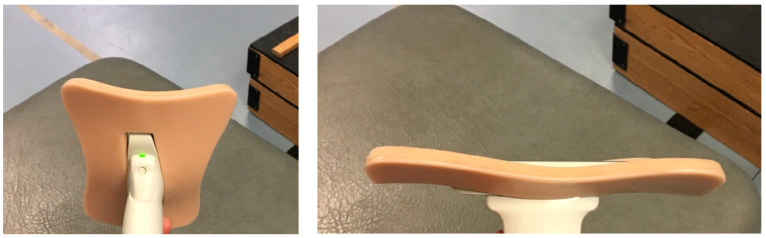
Custom-designed cast, housing a 10 cm ultrasound probe.

The same probe and ultrasound scanner was utilized to collect dynamic ultrasound clips of the BF_LH_ fascicles during the NHE variations for the self-identified dominant leg. A custom designed cast was utilized that was able to securely house the probe (Figure 2). Double-sided adhesive tape and elasticated bandages were used to attach the probe and cast to the posterior thigh. Enough conductive gel was applied to the probe prior to being fixed upon the posterior thigh, where it was fixed in orientation to the guideline to achieve an accurate plane to view the BF_LH_ fascicles.

Three-dimensional motion data for the NHE variations were collected using infrared cameras (Qualisys, Partille, Sweden). Passive retro-reflective markers were placed on each of the lateral portions of the participants’ legs and trunk (lateral malleoli, lateral femoral epicondyles, greater trochanter and acromion process). Motion data were collected at 250 Hz and filtered using a 5 Hz single cutoff frequency filter.

Surface sEMG activity of the contralateral limb BF_LH_ and medial hamstrings was measured for all trials. Participants’ skin was prepared using a standardized process including shaving and alcohol wipes [15]. Electrodes 10 mm in diameter and with a 17.5 mm inter-electrode distance (Noraxon U.S.A. Inc, Scottsdale, AZ, USA) were placed at the mid-point of the BF_LH_ and medial hamstrings, as per SENIAM guidelines. Raw sEMG data were captured at 1500 Hz. Correct electrode placement was confirmed prior to commencing data collection with manual muscle testing (i.e., by asking the participants to voluntarily contract the hamstrings against manual resistance), and minimal crosstalk was visually and physically checked via internal and external rotation of the leg with a 90° knee angle.

**Figure A1:**
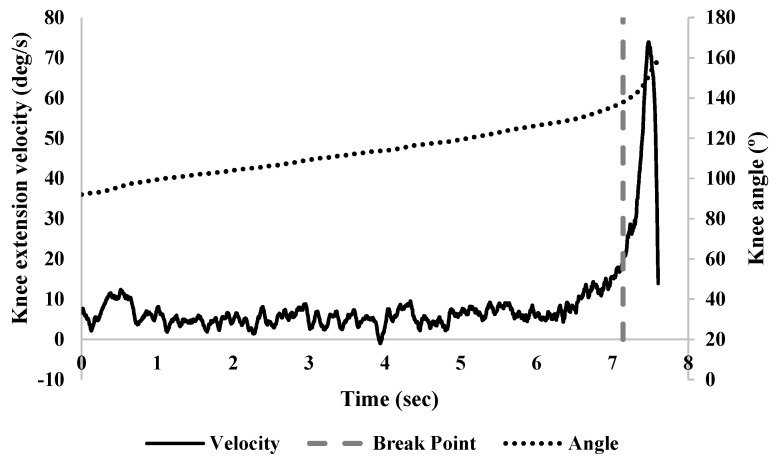
Time–knee extension angle and time–knee extension angular velocity graphs, identifying the moment of break point >20°/s threshold.

Analysis of motion data identified instantaneous hip (lateral femoral epicondyle, greater trochanter and acromion process) and knee (lateral malleoli, lateral femoral epicondyles, greater trochanter) angles and knee angular velocity. Raw data were subsequently exported into a custom-designed Excel spreadsheet (Microsoft, Redwood, WA, USA), and movement onset was identified as the moment participants moved >5° from an average knee angle taken from the first two seconds of data collection. To identify the moment where participants could no longer control the descent (i.e., break point), a knee angular velocity threshold of ≥20°/s was utilized (Figure A1). From the instantaneous hip and knee angles, estimations of BF_LH_ muscle tendon-unit lengths were calculated using regression equations [16]. Kinematic (joint angle and muscle tendon unit length) variables were extracted at the break point, but the break point was also used for time normalization of the NHE within the dynamic ultrasound videos.

Raw sEMG data were high- and low-pass filtered between 10 and 1000 Hz. Processing of the sEMG data was performed with a root mean square filter across a moving average window of 25 ms. Normalization to a perceived maximum value was not performed, as comparisons of sEMG intensity between variations were performed within individuals (i.e., between NHE variations) and not between individuals. Peak sEMG amplitude of both the BF_LH_ and the medial hamstrings was identified across the normalized NHE.

Resting sonograms were analyzed offline with Image J (Version 1.52, Wayne Rasband National Institute of Health, Bethesda, MD, USA). A linear equation (Equation (1)) was utilized to estimate BF_LH_ FL.

FL = L + (h ÷ SIN(β))(1)

Equation (1): fascicle length estimation using the partial measure equation, where L is the observable fascicle length, h is the perpendicular distance between the superficial aponeurosis and the fascicle’s visible end point, and β is the angle between the fascicle and the superficial aponeurosis.

Dynamic ultrasound videos were analyzed using a semi-automated tracking algorithm processed using the UltraTrack MATLAB graphical user interface (MathWorks, Natick, MA, USA). The first ultrasound image of each video sequence, a muscle region of interest and fascicle end points were defined. The muscle region of interest was defined as the area between the superficial and deep aponeuroses of the BF_LH_ muscle that was visible in the ultrasound image. Changes in the BF_LH_ FL were tracked using an optical flow algorithm with affine optic flow extension [17].

Data are presented as mean ± standard deviation (SD); JASP ((version 0.19.0) computer software) was used for all statistical analysis. Normality was assessed by the Shapiro–Wilk statistic. Absolute and relative between-trial reliability of peak measures was assessed by coefficient of variation (CV) percentages and two-way random effects model intraclass correlation coefficients (ICCs), with 95% confidence intervals (CIs) determined for both measures of reliability. Absolute reliability (CV) was interpreted based on the upper-bound 95% confidence intervals as <5% excellent, 5–10% good, 10–15% moderate and >15% poor. A coefficient of multiple correlations (CMCs) with a 95% CI was also performed to analyze the similarity between waveforms by comparing shape, timing and amplitude [18]. The ICC and CMC values were interpreted based on the lower-bound CI as (<0.50) poor, (0.5–0.74) moderate, (0.75–0.90) good and (>0.90) excellent [19].

Repeated measures analysis of variance (RMANOVA) with Bonferroni post hoc corrections was conducted to determine whether there were significant differences in the in vivo mechanics and the kinematic and sEMG changes in performance of the NHE between incline, flat and decline. Hedge’s *g* effect sizes were also calculated to provide a measure of magnitude of the differences in each variable, interpreted in line with previous recommendations, which defined values of <0.35, 0.35–0.79, 0.80–1.5 and >1.5 as trivial, small, moderate and large, respectively [20]. Statistical significance was defined as *p* < 0.05 for all tests.

To identify differences in absolute and relative BF_LH_ FL between the three performance angles, the whole repetition (1–100%) was used as one input using an Excel spreadsheet. Likely differences between NHE performance angle waveforms for absolute and relative BF_LH_ FL changes were determined by plotting the time-normalized average curves for each group, along with the corresponding upper and lower 95% CIs to create upper and lower control limits and identify non-overlapping areas.

## 3. Results

Descriptive and reliability statistics for kinematics are presented in Table 1. Kinematic measures demonstrated moderate–good absolute reliability. Poor–good relative reliability was observed, although measures of MTU length were poor–moderate.

**Figure 3:**
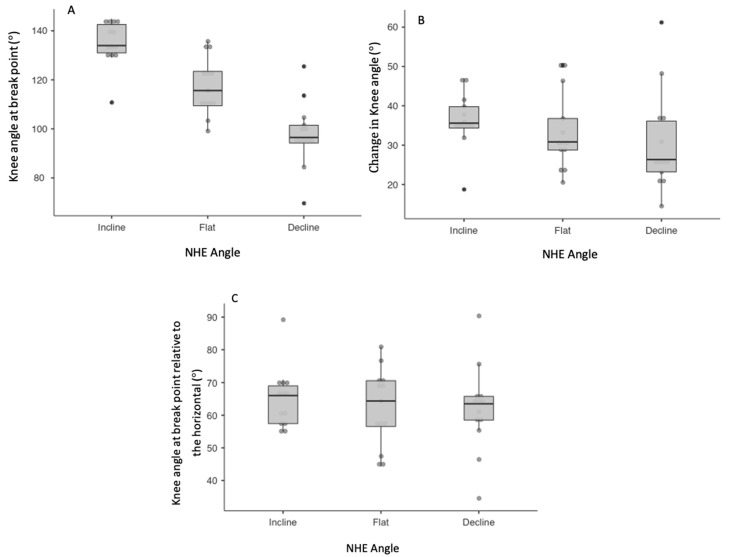
Individual, mean, interquartile range, minimum, maximum and outliers within box-and-whisker plots for the kinematic measures of knee angle. (**A**) Knee angle at break point, (**B**) change in knee angle, (**C**) knee angle at break point relative to the horizontal.

A significant main effect was observed between NHE performance angles (*p* < 0.001). Post hoc analysis revealed significant, moderate-to-large differences for the knee angle at the break point across all performance angles (Figure 3, Table A1), whereas for MTU length at break point there was only a significant, moderate difference between incline and decline variants (Table A1). There were no significant differences observed for change in knee angle or change in MTU length.

Descriptive and reliability statistics for all sEMG measures are presented in Table 2. The absolute and relative reliability of sEMG measures was moderate–good for both the BF_LH_ and ST (Table 2).

No significant main effect was observed between NHE performance angles (*p* = 0.651).

**Figure 4:**
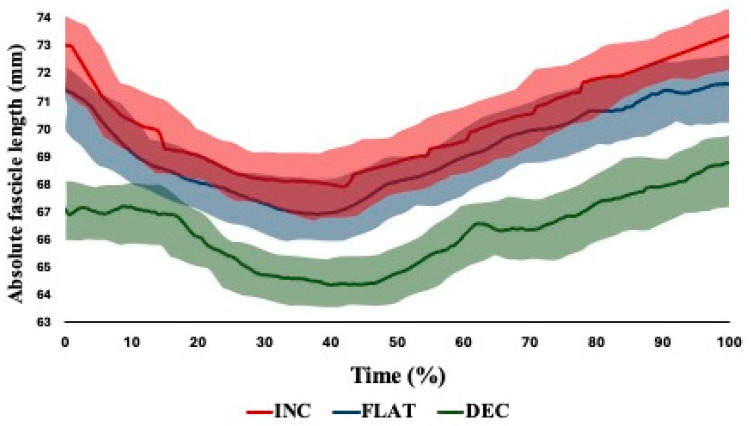
Individual absolute fascicle length waveforms with upper- and lower-bound 95% confidence intervals (shaded) between performance angles (INC = incline; DEC = decline).

**Figure 5:**
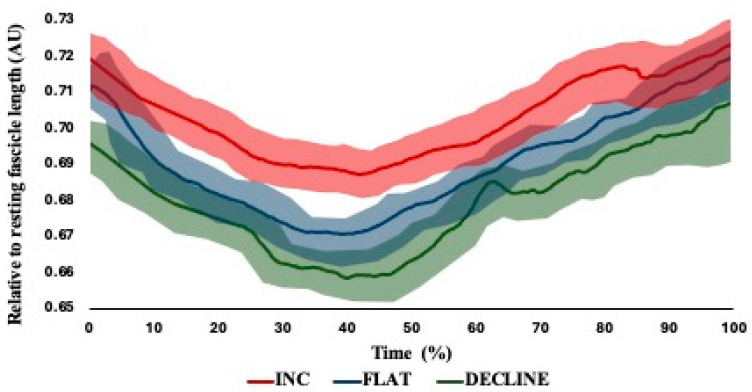
Individual relative fascicle length waveforms with upper- and lower-bound 95% confidence intervals (shaded) between performance angles.

Moderate between-trial reliability was observed across the dynamic ultrasound waveforms for each variation of the NHE (Table 3). Significant main effects (*p* < 0.001) were observed, with significant and large differences between the starting FL, shortest FL, FL at break point and total dynamic FL change between performance angles (Table A2). Likely differences based on the shaded 95% CI were observed between the NHE performance angle for both absolute and relative waveforms between flat and decline NHE performance (Figure 4) and between incline and decline NHE performance (Figure 5).

## 4. Discussion

The incline variation of the NHE resulted in a more active ROM, as hypothesized and in line with previous literature [9]. The current study is only the third study to the authors’ knowledge to image dynamic changes in BF_LH_ FL within the NHE, as with previous works shortening and lengthening was observed [5,6]. FL is impacted by the performance angle, likely as an effect of changes in joint angles and MTU lengths. During the flat and decline variations of the NHE, there was a reduction in knee angle and MTU length at the break point. There were moderate–large increases in knee angle at the break point and small–moderate increases in MTU length for the incline variation in comparison to flat and decline variations. Between the variations, only trivial–small differences were observed between the change in knee angle and the change in MTU length. Additionally, using the break point angle relative to the horizontal as an indicator of gravitational moment, there were trivial-to-small differences, highlighting that the forces experienced may be similar between variation when achieving a break point even at different muscle lengths. Consistent with the work of Sarabon et al. [9] there was decreasing peak muscle activity between variations of the NHE, although it was trivial–small in magnitude.

Training at longer muscle lengths has shown to result in greater positive adaptations in BF_LH_ FL, whereas in contrast, training at a shorter muscle length results in a greater positive adaptation for eccentric hamstring strength [21]. Franchi et al. [22] highlighted the potential of a more pronounced mechanical stretch that could be applied to single sarcomeres during larger ROM tasks, influencing serial sarcomere distribution and eventual architectural adaptations [23]. This may signify that an incline NHE, where the knee joint is in an elevated position in comparison to when attached at the ankles, could lead to larger positive adaptations in BF_LH_ FL in contrast to the traditional NHE or decline variations. It has also been observed that training at a greater muscle length results in similar or greater increases in muscle size [24]. This indicates that utilizing an incline NHE could lead to an increase in hamstring muscle volume, which could be extremely beneficial for athletes who require simultaneous adaptations in muscle size and architectural adaptations, such as youth team sport athletes or aesthetic athletes. When performing the NHE at a longer muscle length, an increase in early torque production has been identified [25]; however, the present study did not measure or assess torque. As a similar break point is achieved during the NHE, the peak torques attained would likely be similar, which is consistent with work by Sarabon and colleagues [9], which is supported by consistency within the gravitational moment (knee angle at break point relative to the horizontal). Therefore, adaptations in eccentric hamstring strength could be similar between the NHE performance angles, as the peak eccentric torques (or gravitational moments, as used in the present study) are identical if a break point is achieved. Practitioners implementing the NHE aims should be increasing eccentric hamstring strength and BF_LH_ FL [1,26,27,28,29].

To the authors’ knowledge, this is the third study to observe hamstring in vivo mechanics during the NHE but only the second to effectively assess changes in hamstring muscle architecture across an entire exercise movement, with previous published works only observing end-stage ROM of the NHE (>85% maximum force) [5]. The difficulties in dynamically imaging hamstring muscle architecture have been explored previously, including field of view, spatiotemporal resolution, transducer design and image quality [5,6,18,29]. Despite these difficulties, the current study found moderate levels of between-trial reliability using both ICCs and CMCs (Table 3); only Van Hooren and colleagues [6] have identified measures of reliability for the vivo previously, but this was based of absolute values of BF_LH_ FL excursion and velocity, where there was good and poor reliability based off the point estimate, respectively. The moderate level of reliability reported also highlights the ability of the semi-automated tracking system to dynamically assess the BF_LH_ during exercise, although Raiteri et al. [5] identified that there was a significant difference between tracking algorithms (UltraTrack vs. pointTrack), with UltraTrack reporting a shorter change in FL during the NHE to a large magnitude. Across all the NHE variations for both absolute and relative FLs, approximately 40% of the ROM involved fascicle shortening (i.e., concentric action), after which there was fascicle lengthening (i.e., eccentric action) (Figure 4 and Figure 5).

Between the three NHE variations across the entire ROM, the incline variation involved a greater absolute and relative FL in comparison to the other variations, with likely differences across the waveform (Figure 4 and Figure 5, respectively). In contrast, the decline NHE variation involved a lower absolute and relative FL across the entire ROM. One explanation for the difference in FLs could be from the initial starting positions—as the incline NHE commences at a more extended knee angle and a greater MTU length, it is unsurprising that the FL would be greater in comparison to the other variations. The greater degree of fascicle lengthening observed in the incline NHE could indicate that the incline NHE maybe preferential when attempting to achieve architectural adaptations of the BF_LH_ through great mechanical stress placed on the fascicles [22]. The results of the present study are consistent with those from previous literature, with similar shortening following by lengthening observed by Van Hooren et al. [6], although in contrast, lengthening in the FL only occurred after the break point, with initial shortening followed by an isometric state where there was very little change in FL. However, this could be explained by the hip flexion that occurs concurrently with knee extension, potentially performing an exercise closer to a quasi-isometric exercise, similar to the razor curl. It is also worth noting that the break point was only identified as the moment of “free fall” by Van Hooren and colleagues [6], which is a subjective measure. Although Raiteri et al. [5] also observed net fascicle lengthening during the NHE, they were only able to assess images once forces had reached >85% peak force within the NHE; therefore, they likely missed the initial shortening prior to reaching the moment of fascicle lengthening. The authors proposed that this could be occurring between 0 and 50% of peak force, which is support by the present research even without an accurate measurement of force being identified and has been suggested to be taken up by elastic components within other muscle groups [30,31]. As the early change or shortening is likely being taken up by elastic components, the eventual lengthening when reaching high forces could be further supporting the suggestion of increased mechanical load placed on the individual sarcomeres. However, the present study only observed the dynamic changes occurring within the BF_LH_ without observing the other muscles within the hamstring group. Within the quadriceps muscle group, dynamic FL changes during eccentric exercise are individual-muscle specific, typically because of different muscle compositions [30,31]. Therefore, it could be speculated that within the hamstring complex during eccentric exercises, there is variety of dynamic FL changes occurring, potentially highlighting the need for a multi-factorial training process.

The sEMG response demonstrated that between NHE performance variations, there were minimal, trivial-to-small differences between NHE variations and the individual muscles response. Sarabon et al. [9] identified a significant, small–moderate decrease in relative activation of the BF and medial hamstrings with increasing slope (or performance angle), which is consistent with the present study, albeit to a smaller magnitude. However, due to the impact of normalizing sEMG amplitudes, any normalization procedure could have influenced the observed result [32]. A normalization procedure was not performed for the present study, as comparisons of sEMG intensity between variations were performed within individuals and not between. Therefore, a more accurate reflection of the task intensity and patterning could be provided from non-normalized sEMG amplitudes, despite impacting the comparisons to Sarabon et al. [9]. The individual variations (Table A2) within sEMG could in fact be the result of individual preferential coordination strategies performed within and between the tasks [33,34], with the potential for variations in force-sharing strategies within and between the muscles of the hamstrings. Although this requires further investigation, investigating this with a reflection of region-specific activation and fiber strain would also be beneficial [35,36,37,38]. Moreover, future research should observe sEMG at the break point and sEMG time waveforms through the entire range of motion. 

The current study is not without limitations. Firstly, the present data only include two-dimensional ultrasound imaging, which fails to capture the complex interactions between transverse and longitudinal muscle strains and the potential rotation around the longitudinal axis that can occur under voluntary actions [18], questioning the validity that two-dimensional imaging provides. Moreover, capturing dynamic FL changes is a difficult task to accomplish, requiring precise application of the ultrasound probe, appropriate fixation ensuring contact with scanning site and issues with muscle bulging during contraction and muscle rotation. As imaging technology improves, further research should look to observe the dynamic fascicle changes that occur across all the hamstring muscles. In addition, assessing dynamic fascicle changes across a range of exercises could be considered crucial to identify potential differences in adaptations. Future dynamic imaging studies should look to group participants by strength, as suggested by Raiteri et al. [5], to be able to understand how individual strength impacts the fascicle dynamics, which could be related to the between-subject differences when using the NHE within athlete training program. This information could highlight differences in strategy of loading, specifically within the NHE, as an individual’s level of strength could alter the velocity and strategy of loading, therefore assisting physical performance coaches and physiotherapists in understanding how to cue the NHE within training for athletes.

## 5. Conclusions

The observed change in NHE starting position, which influenced knee angle and MTU length, could explain the observed differences within the in vivo muscle fascicle changes in the BF_LH_. It could also support the decreased neuromuscular contributions. When compared to a resting FL within the early–mid range of movement (0–40% time), greater fascicle shortening was observed within the decline and flat NHE variations. This is likely due to the contractile components within the BF_LH_ taking up more slack within the elastic component (i.e., distal tendon), which is under less strain during the early stages of the movement. Despite these differences early during the movement, there were no likely differences between variations at the mid–end range (40–100% time). The results of the present study highlight the utility of performance angle manipulation for practitioners, with the ability to regress or progress the NHE with alterations in the lever arm reducing or increasing as required by the hamstrings to resist knee extension within incline or decline variations, respectively. Moreover, all variations resulted in a similar magnitude of fascicle lengthening achieving a similar break point, potentially indicating that a similar positive adaptation in eccentric hamstring strength and BF_LH_ FL may occur.

## Figures and Tables

**Table 1:** Descriptive and reliability statistics for kinematic data across NHE variations.

		Mean (SD)	CV% (95% CI); Descriptor	ICC (95% CI); Descriptor
Knee angle (°) at break point	Incline	135.08 (9.22)	3.67 (2.26–5.08); Good	0.877 (0.626–0.975); Moderate
	Flat	117.67 (11.87)	4.44 (2.73–6.15); Good	0.965 (0.877–0.993); Good
	Decline	98.04 (13.28)	4.64 (2.86–6.42); Good	0.943 (0.809–0.989); Good
Change in knee angle (°)	Incline	36.34 (7.02)	4.89 (3.01–6.77); Good	0.787 (0.628–0.955); Moderate
	Flat	33.44 (9.87)	4.88 (3.00–6.76); Good	0.908 (0.706–0.982); Moderate
	Decline	30.53 (12.65)	4.81 (2.96–6.66); Good	0.951 (0.831–0.991); Good
Knee angle (°) at break point relative to the horizontal	Incline	64.98 (9.22)	6.39 (3.93–8.85); Good	0.822 (0.725–0.985); Moderate
	Flat	61.27 (11.82)	8.80 (5.42–12.18); Moderate	0.912 (0.857–0.963); Good
	Decline	61.94 (13.31)	8.60 (5.29–11.91); Moderate	0.933 (0.849–0.990); Good
Relative MTU length (%) at break point	Incline	100.95 (2.78)	5.61 (3.45–7.77); Good	0.877 (0.626–0.975); Moderate
	Flat	98.64 (2.55)	6.56 (4.04–9.08); Good	0.812 (0.477–0.961); Poor
	Decline	96.77 (3.39)	5.48 (3.37–7.59); Good	0.809 (0.472–0.960); Poor
Change in relative MTU length (%)	Incline	7.73 (0.83)	6.45 (3.97–8.93); Good	0.777 (0.408–0.953); Poor
	Flat	7.63 (2.03)	7.78 (4.79–10.77); Moderate	0.814 (0.482–0.961); Poor
	Decline	7.52 (2.19)	5.29 (3.26–7.32); Good	0.631 (0.176–0.914); Poor

MTU—muscle–tendon unit.

**Table 2:** Descriptive and reliability statistics for electromyography data across NHE variations.

		Mean (SD)	CV% (95% CI); Descriptor	ICC (95% CI); Descriptor
Peak bicep femoris sEMG (μV)	Incline	413.49 (148.75)	6.40 (3.94–8.86); Good	0.857 (0.773–0.889); Good
	Flat	364.70 (124.14)	8.85 (5.45–12.25); Moderate	0.822 (0.680–0.880); Moderate
	Decline	324.98 (109.76)	7.17 (4.41–9.93); Good	0.848 (0.749–0.887); Moderate
Peak semitendinosus sEMG (μV)	Incline	352.45 (121.87)	7.21 (4.44–9.98); Good	0.817 (0.669–0.879); Moderate
	Flat	380.83 (153.72)	6.81 (4.19–9.43); Good	0.808 (0.646–0.876); Moderate
	Decline	380.27 (153.89)	8.97 (5.52–12.42); Moderate	0.830 (0.700–0.882); Moderate

sEMG = electromyography.

**Table 3:** Absolute and relative between-trial reliability for dynamic ultrasound waveforms and mean and SD absolute FL measurements at the starting position, shortest FL, and FL and break point.

	Incline	Flat	Decline
ICC (95% CI) Descriptor	0.677 (0.525–0.709) Moderate	0.717 (0.646–0.786) Moderate	0.679 (0.633–0.725) Moderate
CMC (95% CI) Descriptor	0.669 (0.638–0.701) Moderate	0.701 (0.642–0.740) Moderate	0.672 (0.628–0.715) Moderate
Starting fascicle length (mm)	73.02 ± 0.87	71.43 ± 0.92	67.12 ± 1.16
Shortest fascicle length (mm)	67.89 ± 0.92	66.93 ± 0.37	64.40 ± 1.53
Fascicle length at break point (mm)	73.38 ± 0.93	71.63 ± 0.59	68.76 ± 1.48
Fascicle length excursion (mm)	10.81 ± 1.18	9.22 ± 1.06	7.11 ± 1.52

ICC = intraclass correlation coefficient, CMC = coefficient of multiple correlations, CI = confidence intervals, standard error of the measurement.

## Data Availability

The data presented in this study are available on request from the corresponding author.

## Appendix A

**Table A1:** Pairwise difference between kinematic measures for each Nordic hamstring exercise variation.

		Pairwise Post Hoc	Cohen’s *d* (95% CI)	Effect Size Descriptor
Knee angle (°) at break point	Incline vs. Flat	0.001 ^$^	1.39 (0.14–2.59)	Moderate
	Incline vs. Decline	<0.001 ^$$^	2.82 (1.20–4.38)	Large
	Flat vs. Decline	0.002 ^$^	1.30 (0.06–2.49)	Moderate
Change in knee angle (°)	Incline vs. Flat	0.667	0.29 (−0.81–1.38)	Trivial
	Incline vs. Decline	0.337	0.51 (−0.61–1.61)	Small
	Flat vs. Decline	0.792	0.22 (−0.88–1.31)	Trivial
Knee angle (°) at break point relative to the horizontal	Incline vs. Flat	0.801	0.37 (−0.74–1.46)	Small
	Incline vs. Decline	0.780	0.42 (−0.69–1.51)	Small
	Flat vs. Decline	0.997	0.04 (1.05–1.13)	Trivial
Relative MTU length (%) at break point	Incline vs. Flat	0.090	0.70 (−0.44–1.81)	Small
	Incline vs. Decline	0.006 ^$^	1.14 (−0.07–2.30)	Moderate
	Flat vs. Decline	0.270	0.54 (−0.58–1.64)	Small
Change in relative MTU length (%)	Incline vs. Flat	0.984	0.06 (−1.03–1.15)	Trivial
	Incline vs. Decline	0.945	0.12 (−0.97–1.21)	Trivial
	Flat vs. Decline	0.991	0.04 (−1.05–1.13)	Trivial

MTU = Muscle-tendon unit, ^$^ = *p* < 0.05, ^$$^ = *p* < 0.001.

**Table A2:** Pairwise differences between in-vivo mechanics measures for each Nordic hamstring exercise variation.

		Pairwise Post Hoc	Cohen’s *d* (95% CI)	Effect Size Descriptor
Starting fascicle length (mm)	Incline vs. Flat	0.004	1.78 (0.44–3.06)	Large
	Incline vs. Decline	0.000	3.75 (1.12–5.33)	Large
	Flat vs. Decline	0.000	2.12 (1.08–3.11)	Large
Shortest fascicle length (mm)	Incline vs. Flat	0.007	1.37 (0.12–2.57)	Moderate
	Incline vs. Decline	0.001	2.76 (1.16–4.30)	Large
	Flat vs. Decline	0.001	2.27 (0.81–3.68)	Large
Fascicle length at break point (mm)	Incline vs. Flat	0.001	2.25 (0.79–3.65)	Large
	Incline vs. Decline	0.000	3.74 (1.83–5.60)	Large
	Flat vs. Decline	0.000	2.55 (1.01–4.03)	Large
Total dynamic change in fascicle length (mm)	Incline vs. Flat	0.005	1.42 (0.16–2.63)	Moderate
	Incline vs. Decline	0.001	2.72 (1.13–4.25)	Large
	Flat vs. Decline	0.005	1.61 (0.31–2.86)	Large

MTU = muscle–tendon unit
